# Uninstructed BIAT faking when ego depleted or in normal state: differential effect on brain and behavior

**DOI:** 10.1186/s12868-016-0249-8

**Published:** 2016-05-03

**Authors:** Wanja Wolff, Sebastian Schindler, Christoph Englert, Ralf Brand, Johanna Kissler

**Affiliations:** Department of Sport Science - Section Sport Psychology, University of Konstanz, Universitätsstr. 10, 78457 Constance, Germany; Sport and Exercise Psychology, University of Potsdam, Im Neuen Palais 10, 14469 Potsdam, Germany; Department of Psychology, University of Bielefeld, Universitätsstr. 25, 33615 Bielefeld, Germany; Center of Excellence Cognitive Interaction Technology (CITEC), University of Bielefeld, Inspiration 1, 33619 Bielefeld, Germany; Department of Educational Psychology, University of Bern, Fabrikstr. 8, 3012 Bern, Switzerland

**Keywords:** EEG/ERP, Implicit association test (IAT), Faking, Deception, Ego depletion, Cognitive control

## Abstract

**Background:**

Deception can distort psychological tests on socially sensitive topics. Understanding the cerebral processes that are involved in such faking can be useful in detection and prevention of deception. Previous research shows that faking a brief implicit association test (BIAT) evokes a characteristic ERP response. It is not yet known whether temporarily available self-control resources moderate this response. We randomly assigned 22 participants (15 females, 24.23 ± 2.91 years old) to a counterbalanced repeated-measurements design. Participants first completed a Brief-IAT (BIAT) on doping attitudes as a baseline measure and were then instructed to fake a negative doping attitude both when self-control resources were depleted and non-depleted. Cerebral activity during BIAT performance was assessed using high-density EEG.

**Results:**

Compared to the baseline BIAT, event-related potentials showed a first interaction at the parietal P1, while significant post hoc differences were found only at the later occurring late positive potential. Here, significantly decreased amplitudes were recorded for ‘normal’ faking, but not in the depletion condition. In source space, enhanced activity was found for ‘normal’ faking in the bilateral temporoparietal junction. Behaviorally, participants were successful in faking the BIAT successfully in both conditions.

**Conclusions:**

Results indicate that temporarily available self-control resources do not affect overt faking success on a BIAT. However, differences were found on an electrophysiological level. This indicates that while on a phenotypical level self-control resources play a negligible role in deliberate test faking the underlying cerebral processes are markedly different.

**Electronic supplementary material:**

The online version of this article (doi:10.1186/s12868-016-0249-8) contains supplementary material, which is available to authorized users.

## Background

Test faking is a widespread problem especially when the content of human feelings and thought shall be explored [1]. Socially sensitive topics (e.g., stereotyping, racism, doping) are particularly vulnerable to faking. For example, athletes’ attitudes toward doping that were assessed via self-report measures have shown to be affected by deceptive responses [2]. These authors illustrated that athletes’ self-reported doping attitudes should be considered severely skewed towards the socially desired ‘no to doping in sports’. Reaction-time based indirect tests like the Implicit Association Test [IAT; 3] have been introduced with the promise of being more robust towards deception attempts [4]. There is solid evidence that IAT’s generally outperform traditional self-report measures when socially sensitive topics need to be assessed [5, 6]. Recently a doping Brief IAT (BIAT) has been developed that showed to be a valid predictor of biochemical doping test results [7]. One reason for this might be that compared to direct tests IAT’s are thought to be superior in hiding the true measurement goal (i.e., participants are not directly asked about their attitude towards a certain topic). Typically, these tests are presented as lexical sorting tasks on a computer, where two concepts (one target and one evaluative) are mapped on the same response key of the keyboard and participants are requested to respond as fast as possible. The task is easier and reaction times are faster when the two concepts sharing the same response key (e.g., doping and dislike) are closely associated than when they are not associated (e.g., doping and like).

However, even IAT’s can be faked to some extent; even more if participants had the opportunity to familiarize themselves with the procedure at least once [8]. Research indicates that participants who were provided with an explicit faking strategy were more successful at faking than participants who were simply instructed to find a way to ‘trick the test’ [8–12]. One major reason why faking IATs has sparked scientific interest is very practical: Test takers whose motivation to disguise their true attitude is high (for example towards doping in sports) will most likely begin to think about (and try) deception strategies. However, a recent study finds that in more realistic setting, i.e., when participants were only implicitly incentivized to fake doping attitude tests, faking occurred on self-reported measures but not on the BIAT [6]. Research is needed to identify the cognitive processes that facilitate (or inhibit) participants’ faking success. Studying the electrophysiological correlates underneath these processes helps to understand how (and drawing upon which resources) participants fake such response time-based tests. Using electroencephalography’s (EEG) very high time resolution of event-related potentials (ERPs) enables to investigate very early cortical processes during faking.

### Cortical processes of test faking

So far, most ERP research on test faking focused on forced choice test formats [13–16]. Some studies found larger very early frontal negativity [16] and an occipital positivity [14] for faking. Such early effects [16] have also been attributed partly to the blocked designs used in this research where participants had the opportunity to prepare themselves for giving an appropriate faked answer [17]. Consistently, for designs using an equal number of stimuli in the faking and non-faking condition, a reduced late positivity has been found, starting at the P300 [13–16, 18, 19].

This is thought to reflect a form of cognitive control, where participants have to exert self-control [14]. Recently ERP patterns were investigated while participants that were provided with an effective faking strategy faked a validated doping brief implicit association test [BIAT, 17]. Here, significant differences were found between faking and non-faking at early and late time points. An enlarged early frontal negativity and occipital positivity was found as well as a decreased P300/LPP component [17]. Source analyses revealed significantly more activity in the right inferior frontal gyrus and the bilateral temporo-parietal junction for faking compared to baseline blocks. Among other processes the right inferior frontal gyrus is involved in memory and motor inhibition [20–23] which suggests an inhibition of a prepotent motor response to the target stimulus. The enhanced activity for faking in the temporo-parietal junction was thought to reflect the monitoring on faking and faking success. For these middle temporal/occipital regions enhanced activity is also found in an functional magnetic resonance imaging (fMRI) study on lying [24]. To conclude, the act of suppressing a predominant response tendency (i.e., non-faking response) and substituting it with an experimentally required response (i.e., faking response) is an act of inhibitory cognitive control or self-control [25]. To our current knowledge there seems to be no specific ‘faking’ component visible in ERPs [14]. Individual differences in temporarily available self-control resources might affect how the cerebral response to such faking demands is and therefore partly explain the lack of a specific ‘faking’ component.

### Ego depletion as a cognitive moderator of the cerebral faking response

According to the strength model of self-control [26], the ability to volitionally inhibit predominant response tendencies is an act of self-control which is relies on the temporary availability of self-control strength. Baumeister and colleagues argue that all self-control acts e.g., emotion regulation [27] and attention regulation [28] are energized by one global resource, i.e., a metaphorical strength with limited capacity. Self-control strength can be depleted after a primary self-control act (i.e., *ego depletion*) and is not replenished immediately. Thus, in a state of ego depletion, less self-control strength is available for subsequent acts of self-control. This can negatively affect performance on tasks that require self-control. Previous research has shown that the ability to suppress unwanted thoughts or attitudes (e.g., stereotypes) depends on self-control strength [29]. Therefore, we assume that faking also requires self-control strength. It has to be noted, that the strength models’ propositions have been questioned lately [30]. Specifically, it has been argued that the estimated ego-depletion effect size might have been overestimated [30]. A Registered Replication Report is currently underway to investigate the size of the ego-depletion effect and to shed further light on this phenomenon (https://osf.io/jymhe/).

Still, neuroscientific research has provided some support for the strength model of self-control. For instance, depleted participants displayed weaker error related negativity (ERN) signals while they performed a self-control task [31]. Although neuroscientific findings of depletion effects have been found to be somewhat inconsistent [32], one expectation of ego depletion effects is a reduction of activity within some parts of executive networks [32]. Specifically, Friese et al. [33] found that ego depletion was associated with an activity decrease in the right lateral prefrontal cortex, an area which is responsible for the effortful implementation of control.

### The present research

Our study is motivated by the idea that in test faking, participants have to voluntarily inhibit a predominant or at least more ‘automatic’ response tendency (i.e., telling the truth). It is thus reasonable to assume that faking draws upon self-control resources. Faking research has not been linked with research on ego depletion yet and no study addressed how neurophysiological faking correlates differ as a function of the available self-control resources.

We hypothesize that ego depletion leads to a decreased ability to exert cognitive control [34]. We thus expect a distinctive ERP response for IAT faking under ego depletion compared to IAT faking when participants are not depleted. Further, we aim at extending previous findings on instructed BIAT faking by investigating the ERP response for non-depleted BIAT faking when participants have to search for an effective faking strategy themselves. For faking without an explicit strategy provided we expect similar ERP differences as for faking with an explicit strategy [17]. Namely, we expect an early frontal negativity and occipital positivity, and a subsequent decrease in the late components for faking. Further, in source space we expect more activity for faking in the right-inferior frontal gyrus and the bilateral temporoparietal junction (TPJ). Finally, we expect that for faking under ego depletion these differences should be reduced or even absent.

## Methods

In line with suggestions of Simmonset al. [35] “we report how we determined our sample size, all data exclusions, all manipulations, and all measures in the study.” All data are uploaded in Additional file 1, Additional file 2 and Additional file 3.

### Participants

Twenty-four participants were recruited at the University of Bielefeld. We aimed for the same sample size as in the previous study on IAT faking [17] in order to increase comparability of results and because both studies are closely comparable in the experimental design. They gave written informed consent according to the Declaration of Helsinki and the study was approved by the ethics committee of the University of Bielefeld. Two participants were excluded due to excessive artifacts (both >50 % bad trials) leaving 22 participants for final analyses. These 22 participants (15 females) were 24.23 years old on average (*SD* = 2.91, *Min* = 21, *Max* = 31) and had normal or corrected to normal vision. Screenings with the German version of the Beck Depression Inventory and the State Trait Anxiety Inventory [36, 37], revealed neither clinically relevant depression symptoms (*M* = 3.55, *SD* = 3.54) nor pathological anxiety scores (*M* = 34.73; *SD* = 5.12). No participant reported previous or current diagnosed mental or neurological disorders. One subject was left handed.

### Design/procedure

**Fig. 1:**
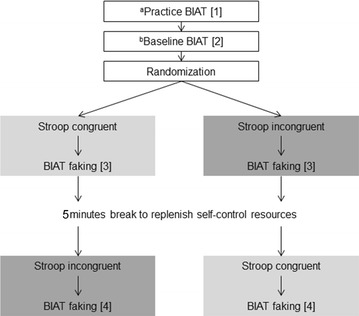
The experimental design. Participants completed a total of four BIAT‘s. The first two BIAT‘s were completed with the standard instructions and the last two BIAT‘s were completed with the instruction to fake a negative doping attitude. Faking under ego depletion was operationalized by an incongruent Stroop preceding a BIAT (*dark grey area*) and ‘normal’ faking was operationalized by a congruent Stroop task preceding a BIAT (*light grey area*). ^a^The practice BIAT consisted of 20 discrimination trials; and 20 trials in each of the doping + like and the doping + dislike blocks. ^b^The discrimination block was removed for the subsequent BIAT‘s and the doping + like and doping + dislike blocks were increased to 40 trials each

The participants’ ability to fake a negative doping attitude in an ego depleted versus non-depleted state was tested in a counterbalanced within-group (repeated measures) experimental design (Fig. 1). All participants completed a practice doping BIAT in order to familiarize themselves with the task [38]. This BIAT was excluded from subsequent analyses. As a baseline measure of their doping attitude, participants then completed another doping BIAT. They were then randomized to either the ‘ego depletion’ or to a ‘non-depletion’ condition and completed the respective tasks. Immediately after the manipulation both groups were asked to complete another doping BIAT and were instructed to fake the test in a way that they appeared strongly anti-doping. Participants were asked to find their own way to “trick” the test (An effective way to trick the test is to deliberately delay the response in the *doping* + *like* block and to respond with full effort in the *doping* + *dislike* block [8, 11, 12, 17]). Research has shown that significant resource replenishment occurs within 5 min [39]. Therefore, after a break of 5 min the order was reversed: Participants who were depleted in the first sequence of the experiment were not depleted in the second sequence (and vice versa). Again, all participants were asked to fake the final doping BIAT in a way that they appeared strongly anti-doping. Manipulation checks whether or not participants perceived the depletion task as more depleting than the control condition were performed before both BIAT measurements. All experimental stimuli were programmed and run using Inquisit 3.0 experimental software (www.millisecond.com).

### Measures

#### Biat

Doping attitudes were assessed with a validated pictorial doping BIAT [40]. The practice BIAT consisted of a discrimination block (20 trials) where participants learned to discriminate the doping stimuli from the non-focal health food stimuli using the ‘R’ and ‘I’ keys on the computers keyboard. Then the compatible block (*doping* + *dislike*, 20 trials) was presented, followed by the incompatible block (*doping* + *like*, 20 trials). The order of compatible and incompatible blocks was counterbalanced between participants to avoid positioning effects. In the following BIAT’s, the discrimination block was removed and compatible and incompatible blocks were expanded to 40 trials each. This BIAT is identical to the one introduced by Brand, Heck and Ziegler [40] with the exception that (a) we expanded the blocks of interest to 40 trials to get an adequate number of trials per cell for ERP averaging and (b) set the inter-trial-interval to 1000 ms in order to avoid introducing artifacts into the EEG measure.

Pictures of syringes, ampules, and pills represented the focal ‘doping’ concept; pictures of apples, vegetables, and cereal stood for the ‘health food’ concept. The ‘like’ and ‘dislike’ concepts were represented by positive and negative Emoticons. D-scores are calculated according to the D4 algorithm such that negative scores represent a negative attitude towards doping [41]. This means that reaction times of error trials, and those exceeding 10,000 ms are deleted and replaced by an error value (average reaction time of this participant in all correct trials of the block plus an error penalty of 600 ms; mere elimination of error trials would have a negative impact on the reliability of the test).

#### Ego depletion manipulation

A computerized Stroop task [42] was used to induce ego depletion. This task has been frequently applied to experimentally manipulate self-control strength [43, 44]. In this task the participants see a color word on a computer screen. The font color in which the word is written either does match its semantic meaning (i.e., congruent trial; e.g., “blue” written in blue color) or it does not (i.e., incongruent trial; e.g., “blue” written in red color). Participants have to name the font color and not the semantic meaning of the color word. This requires the exertion of self-control as one has to volitionally override the automatic tendency to name the color word [44].

In our study participants indicated the font color of each word by pressing the respectively labeled key on a computer keyboard as fast and accurate as possible. All participants completed 30 practice trials (15 congruent, 15 incongruent) to get familiarized with the task first. Then, participants in the ego depletion condition performed the depleting Stroop task, which consisted of 40 congruent and 40 incongruent trials (order randomized). Participants in the non-depletion condition performed the non-depleting version of the Stroop task which consisted of 80 congruent trials (order randomized).

#### Manipulation check and control variables

In order to assess the degree of self-control participants had to exert while working on the task, participants answered three items (e.g., “How mentally exhausting did you find the Stroop task?”; after depletion: α = 76; after non-depletion: α = .51). To control for differences in motivation participants answered three items (e.g., “How motivated were you to do well in the Stroop task?”; after depletion: α = 90; after non-depletion: α = .97). All items had to be answered on a 7-point Likert-type scale with answers ranging from 1 = *not at all* to 7 = *very much*.

As mood can affect performance on self-control tasks it is routinely controlled for in self-control research [e.g., 43, 45]. We administered the German version of the Positive and Negative Affect Schedule [PANAS; 46]. Participants indicated their positive affect (10 items; e.g., “strong”; after depletion: α = 89; after non-depletion: α = .89) as well as their negative affect (10 items; e.g., “anxious”; after depletion: α = 75; after non-depletion: α = .63). Items had to be answered on a 5-point Likert-type scale with answers ranging from 1 = *not at all* to 5 = *very much*.

Indicating that our ego depletion manipulation worked, participants perceived the depletion task to be more depleting compared to the control task [*M*_depletion-non depletion_ = 0.64; *t*_(20)_ = −2.54, *p* = .019, *d* = 0.56; 95 % CI +0.11 to +1.16], but did not differ in their motivation to perform well in it, *p* = .569. There were no differences in positive or negative affect, all *p*’s > .20.

### EEG recording and preprocessing

EEG signals were recorded from 128 BioSemi active electrodes (www.biosemi.com) with a sampling rate of 2048 Hz. Biosemi uses a Common Mode Sense active electrode (CMS) and a Driven Right Leg passive electrode (DRL) as two as ground electrodes. Four additional electrodes measured horizontal and vertical eye-movement.

Pre-processing and statistical analyses were done using SPM8 for EEG (http://www.fil.ion.ucl.ac.uk/spm/). SPM provides a unitary framework for the analysis of neuroscience data acquired with different technologies, including EEG [47, 48]. In a first step, data were offline re-referenced to average reference. To identify artifacts caused by saccades (horizontal) or eye blinks (vertical), virtual channels were created from the electrooculographic electrodes and then subtracted from the EEG. Data were then downsampled to 250 Hz and band-pass filtered from 0.166 to 30 Hz with a fifth-order zero phase Butterworth filter. Filtered data were segmented from 100 ms before stimulus onset until 1000 ms after stimulus presentation. 100 ms before stimulus onset were used for baseline-correction. Automatic artifact detection was used for trials exceeding a threshold of 150 µV. Data were averaged, using the robust averaging algorithm of SPM8, excluding possible further artifacts. The idea of robust averaging is that for each channel and each time point outliers are down-weighted. An advantage of this approach is that clean averages can be calculated without having clean trials as artifacts are supposed to not consistently overlap and distort only parts of the trials. We used the recommended offset of the weighting function, which preserves approximately 95 % of the data points drawn from a random Gaussian distribution [48]. Overall, 8.10 % of all electrodes were interpolated by the recorded activity of all other channels by spherical spline interpolation [49, 50] and 18.85 % of all trials were rejected, leaving on average 32.46 trials per block.

Source reconstructions of the generators of significant ERP differences were generated and statistically assessed with SPM8 for EEG (http://www.fil.ion.ucl.ac.uk/spm) following recommended procedures. First, a realistic boundary element head model (BEM) was derived from the SPM’s template head model based on the MNI brain. The standard coordinates for all electrode positions then were transformed to match the template head, which is thought to generate reasonable results even when individual subjects head differ from the template [48]. To this aim, average electrode positions as provided by BioSemi were co-registered with the cortical mesh template for source reconstruction. Group inversion [51] was computed and the multiple sparse priors algorithm implemented in SPM8 was applied. This method allows activated sources to vary in the degree of activity, but restricts the activated sources to be the same in all subjects [51]. Compared to for single subjects, this is thought to result in superior source estimation. For source reconstruction, frequency contents between 0.166 and 30 Hz were analyzed [48]. For each analyzed time window, three-dimensional source reconstructions were generated as NIFTI images. These images were smoothed using an 8 mm full-width half-maximum kernel (voxel size = 2 mm × 2 mm × 2 mm). Since we had to use all sensors for our source estimation [52], it has to be noted, that the number of interpolated electrodes might also have influenced our source estimation results.

### Statistical analyses

#### BIAT results

In order to test whether participants differed in manifest faking success as a function of experimental condition, a repeated measures ANOVA (condition: baseline, ‘normal’ faking and ego depletion faking) was calculated to investigate main effects for the resulting *D*-scores. Planned contrasts (pairwise comparisons) were used to investigate significant effects’ (*p* < .05) directions of differences.

#### EEG data analyses

**Fig. 2:**
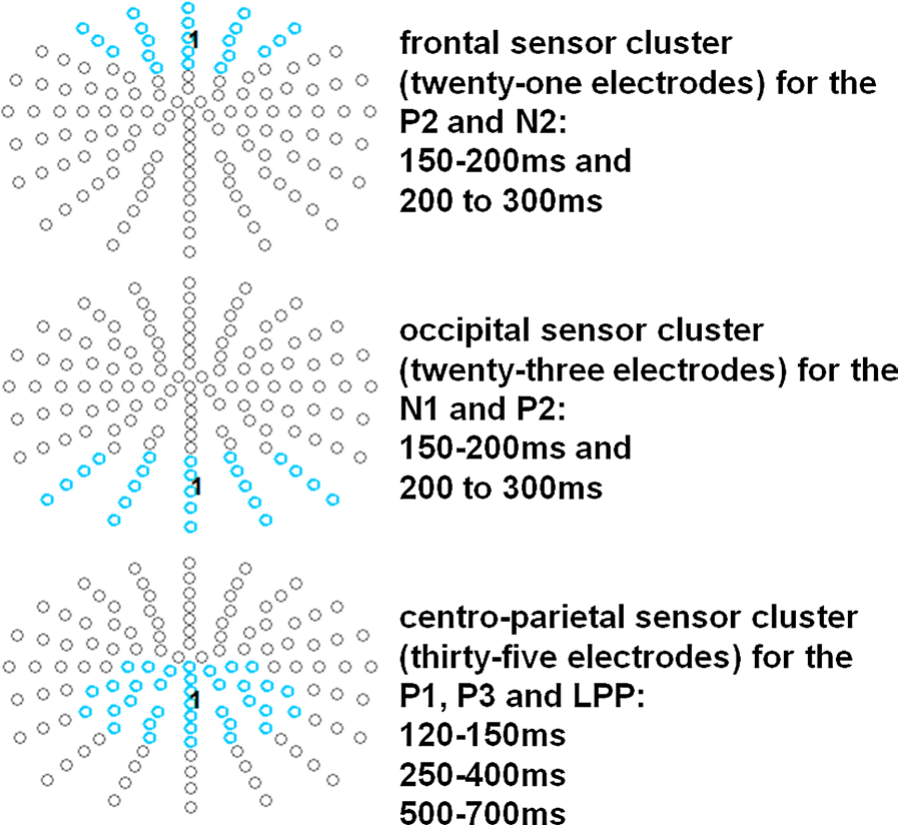
Selected electrode clusters for all investigated components

EEG scalp-data were analyzed with EMEGS [http://www.emegs.org/; 53]. For statistical analyses 2 (block: doping + like vs. doping + dislike) × 3 (condition: baseline vs. ‘normal’ faking vs. ego depletion faking) repeated measures ANOVAs were set-up to investigate interaction effects in time windows and electrode clusters of interest. Whenever Mauchlys Test of Sphericity was violated, Greenhouse-Geisser corrections of the degrees of freedom were performed. Effect sizes for repeated measures were calculated for all effects [54]. Time windows and electrode clusters were segmented similar to Schindler, Wolff and colleagues [17]. Time windows were segmented from 150 to 200 ms to N1/P2 effects, from 200 to 300 ms to investigate P2/N2 effects and from 250 to 400 ms to investigate P3 effects and from 500 to 700 ms to investigate LPP effects. However, the topographical information about significant interactions between block and condition were plotted (see Additional file 4: Figure S1), and an additional time window from 120 to 150 ms was segmented to investigate parietal P1 effects. The selected electrode clusters for all investigated components are displayed in Fig. 2.

Statistical tests for source estimations were calculated for significant scalp interaction time windows. In source space, within significant interactions, post hoc tests were calculated between the doping + like and doping + dislike block for each condition. A threshold of *p* < .005 [e.g., see 55–57] with a minimum of 25 significant voxels [56, 57] was applied. The identification of involved brain regions was performed using the AAL atlas [58].

## Results

### Behavioral results

**Fig. 3:**
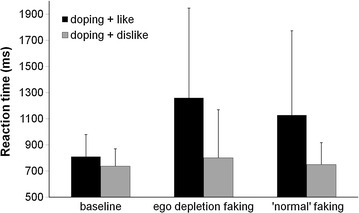
Mean reaction time in each block depicted for each condition. The D-score reflects the standardized mean difference between the *doping* + *like* and the *doping* + *dislike* blocks.* Error Bars* represent standard deviations

Faking in both the ‘normal’ and the depleted faking condition is most likely to occur by responses slowing on the doping + like block and normal performance in the doping + dislike block. However, since participants were not given an explicit strategy, we cannot be sure what strategy they used, or if they used a strategy at all. However, supporting that our faking instruction yielded the desired effect, participants’ response times in the BIAT’s ‘doping + like’ block were slower when they were asked to try faking a negative attitude to doping compared to the baseline BIAT (see Fig. 3). A repeated measures ANOVA on the participants’ raw reaction times revealed a significant interaction between condition and block [*F*_(1.46, 30.62)_ = 9.18, *p* = .002, η_p_^2^ = 304]. Within baseline condition no differences between the doping + like and doping + dislike were found (*p* = .291). In contrast, slower responses were given in the doping + like block both for ‘normal’ faking (*p* = .011) and depleted faking (*p* = .002).

For the participants’ D-scores, similarly a repeated measures ANOVA showed that participants were successful at faking a negative doping attitude on the doping BIAT [*F*_(2, 42)_ = 13.04, *p* < .001, η_p_^2^ = .383]. Planned simple contrast analyses showed that the D-scores in the depletion condition and in the non-depletion condition were significantly higher than baseline D-scores, [*F*_(1, 21)_ = 18.40, *p* < .001, partial η^2^ = .467], and [*F*_(1, 21)_ = 8.57, *p* = .008, η_p_^2^ = .290] respectively. This indicates that our manipulation worked and participants were able to fake an artificially negative attitude to doping. Interestingly, a comparison between the two faking conditions showed a more negative D-score under ego depletion compared to ‘normal’ faking [*F*_(1, 21)_ = 7.85, *p* = .011, partial η^2^ = .272].

### EEG results

#### Centro-parietal sensor cluster

**Fig. 4:**
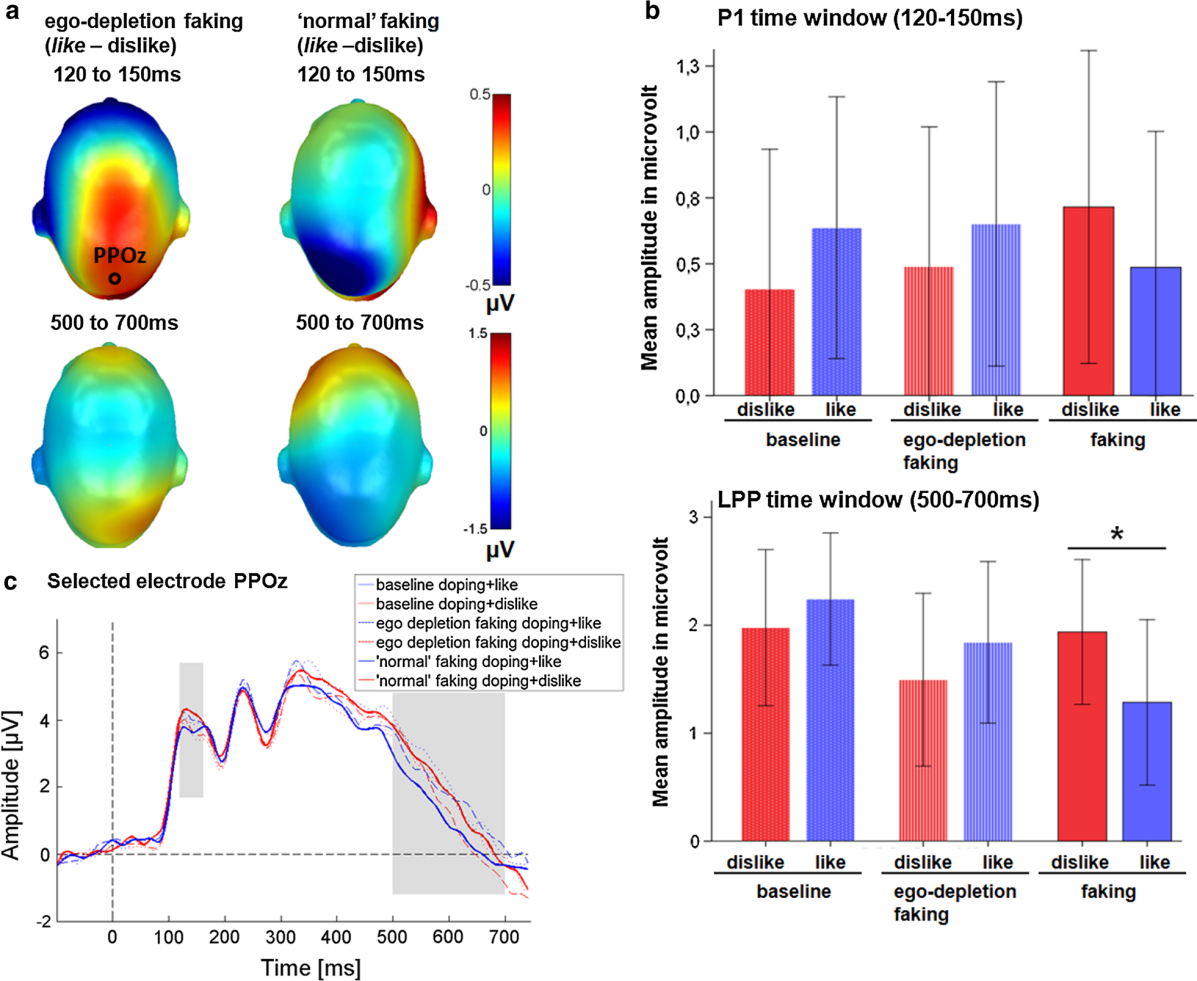
Results for the P1 and LPP time windows over parietal regions. **a** Difference topographies (*doping* + *like*-doping + dislike) for the faking conditions: *blue color* indicates more negativity and* red* color more positivity for the supposed faking block. **b** Mean amplitudes in microvolt over the centro-parietal electrode cluster for the P1 and LPP.* Error bars* represent ±2 times the standard error of the mean. **c** Selected electrode PPOz displaying the time course over parietal sites

Over centro-parietal locations, a significant interaction between (block: doping + like vs. doping + dislike) and condition (condition: baseline, ego depletion faking, ‘normal’ faking) was found already at the P1 [*F*_(2,42)_ = 6.00, *p* = .005, η_p_^2^ = .222; see Fig. 4]. However, post hoc tests did not find significant differences within any condition. In tendency, the doping + like of the ‘normal’ faking condition elicited a larger negativity compared to the doping + dislike block [*M*_*doping*+*like*-doping+dislike_ = −0.23, *t*_(1,21)_ = −1.49, *p* = .151, *d* = −0.18, 95 % CI −0.55 to +0.09], while for the depletion doping + like block a slightly larger positivity was found for this comparison [*M*_doping+like-doping+dislike_ = 0.16, *t*_(1,21)_ = 1.43, *p* = .168, *d* = 0.13, 95 % CI −0.07 to +0.40]. Finally, for the baseline condition a similarly larger positivity was found for the doping + like block [*M*_doping+like-doping+dislike_ = 0.23, *t*_(1,21)_ = 1.93, *p* = .068, *d* = 0.19, 95 % CI −0.02 to +0.48].

Regarding the late stages, in the P3 time window (250–400 ms), a trend for a significant interaction between block and condition was found [*F*_(2,42)_ = 2.63, *p* = .084 η_p_^2^ = .111]. This interaction became significant in the LPP time window (500–700 ms; see Fig. 4; *F*_(2,42)_ = 5.06, *p* = .011 η_p_^2^ = .194). Post-hoc tests showed, that at this time window a significant decreased LPP was observed for the doping + like block of the ‘normal’ faking condition compared to the doping + dislike block [*M*_*doping*+*like*-doping+dislike_ = −0.65, *t*_(1,21)_ = −2.16, *p* = .043, *d* = −0.39, 95 % CI −1.28 to −0.02]. In contrast, there were no differences between the two blocks in the ego depletion faking condition [*M*_doping+like-doping+dislike_ = 0.35, *t*_(1,21)_ = 1.32, *p* = .201, *d* = 0.19, 95 % CI −0.20 to +0.89]. Finally, no differences could be found between the two blocks of the baseline BIAT [*M*_*doping*+*like*-doping+dislike_ = 0.27, *t*_(1,21)_ = 1.14, *p* = .265, *d* = 0.17, 95 % CI −0.22 to +0.75].

#### Frontal sensor cluster

Between 150 and 200 ms, over frontal regions, no interaction was found for the frontal P2 [*F*_(2,42)_ = 0.53, *p* = .592, η_p_^2^ = .025]. For the subsequently occurring N2, between 200 to 300 ms, again no significant block by condition interaction was found [*F*_(2,42)_ = 0.51, *p* = .542, η_p_^2^ = .029]. However, descriptively the expected negativity for the supposed faking block (doping + like) was most pronounced for the ‘normal’ faking condition (*M*_*doping*+*like*-doping+dislike_ = −0.64 µV) compared to ego depletion faking (*M*_*doping*+*like*-doping+dislike_ = −0.28 µV) and baseline condition (*M*_doping+like-doping+dislike_ = −0.25 µV).

#### Occipital sensor cluster

Over occipital sensors, no interaction between block and condition occurred regarding the occipital N1 [150–200 ms; *F*_(2,42)_ = 0.04, *p* = .964, η_p_^2^ = .002], as well as the occipital P2 [200–300 ms; *F*_(2,42)_ = 0.44, *p* = .650, η_p_^2^ = .020].

### Source analyses

**Fig. 5:**
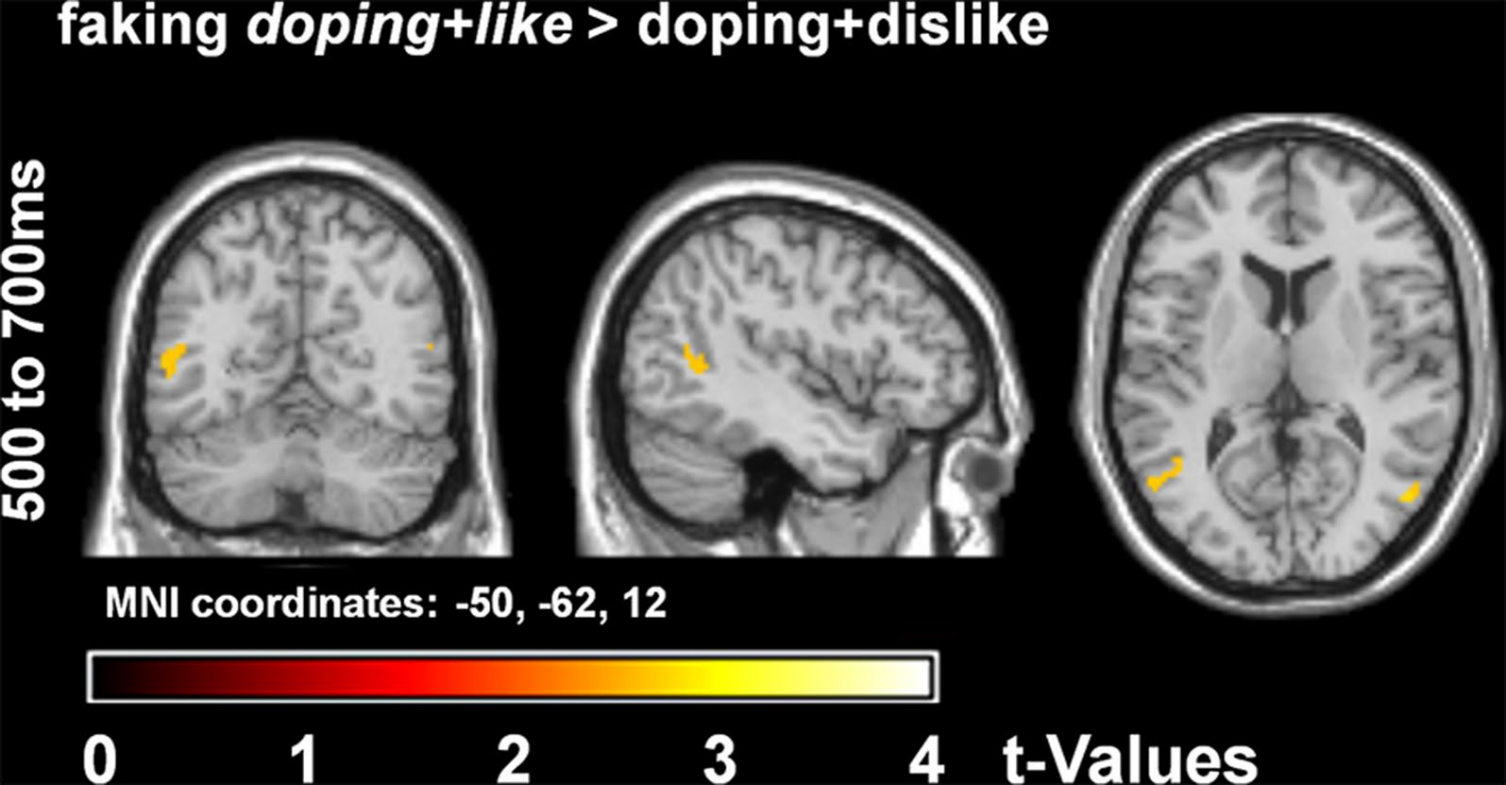
Significant differences in source activity for ‘normal’ faking (*t*-contrasts). The faking block is displayed in italics. In the time window from 500 to 700 ms, more activity was be observed over the bilateral middle temporal gyri/temporoparietal junction for faking

Source analyses were calculated for the significant interaction effects in scalp space. However, no differences in differences for cortical generators could be observed for the P1 interaction. In contrast, in the LPP time window (500–700 ms) a significant interaction was also found in source space (see Fig. 5; Table 1).

**Table 1:** Source analyses for significant scalp effects

No. of sig. voxels per cluster	Peak *F*_(2/126)_	Peak *p* (unc)	MNI space coordinates			AAL
			x (mm)	y (mm)	z (mm)	Area label
Interaction block*condition						
63	6.37	=.002	52	−70	8	Middle occipital R
115	6.10	=.003	−52	−66	12	Middle temporal L
Post-hoc ‘normal’ faking *doping* + *like* > doping + dislike						
29	2.81	=.003	50	−70	10	Middle occipital R
91	2.69	=.004	−54	−64	8	Middle temporal L

Differences between the doping + like and doping + dislike block were calculated within significant interaction terms

No. of sig. voxels per cluster = number of significant voxels for each cluster. unc = uncorrected *p* value. Each cluster may exhibit more than one peak, while only the largest peak is displayed. Peak coordinates are displayed in MNI space (x, y and z). The identification of area labels for each peak was performed using the AAL-atlas. R/L = right or left hemisphere. The faking block is displayed in italics

Post-hoc tests showed that enhanced activity was found for the supposed faking block (*doping* + *like*) in the ‘normal’ faking condition. For the faking block, enhanced activity was found in the bilateral middle temporal gyri/temporo-parietal junction. No differences were found for the baseline condition and the ego depletion faking condition.

## Discussion

Our studies goal was twofold: First, we were interested in the ERP correlates of IAT faking when participants were not provided with an effective faking strategy. Second, we assessed how the ERP’s of faking differed when participants were high or low (i.e., ego depleted) in temporarily available self-control resources.

Behaviorally, participants were able to fake a more negative D-score in both faking conditions compared to the baseline. Somewhat surprisingly, the D-score under ego depletion was even more negative compared to ‘normal’ faking. Regarding ERPs, we found a significant interaction between block and condition already for the parietal P1 component. Post-hoc tests did not reveal significant differences within the respective conditions. However, for the supposed faking block in the ‘normal’ faking condition a descriptively smaller P1 was elicited, while for both the ego depletion faking condition and the baseline condition the reverse pattern was found. For such early components, differences are rarely found for perceptually identical stimuli. This might indicate that faking influences already very early time points, channeling later faking related cognitive processes. Of course one should be aware, that we did not expect this interaction and therefore such early effects need to be replicated.

Nevertheless, it is interesting that the pattern of results matches those found at the late time window (see Fig. 4b). In the P300 time window, there was only a trend for an interaction between block and condition. Importantly, in the later occurring LPP time window the interaction term became significant. Within the faking condition there was a significantly decreased amplitude for the supposed faking block (*doping* + *like*) in the ‘normal’ faking condition. The present data show that for uninstructed ‘normal’ BIAT faking the decrease in the late parietal amplitudes is similar to instructed BIAT faking [17]. Further, this pattern seems to be present already at the P1. We previously noted, that LPP modulations seem to be preceded by earlier sensory differences, but are better predictors of IAT performance [59]. In contrast, post hoc tests did not reveal differences between baseline or ego depletion blocks. For these conditions it seems that the doping + like block elicited larger amplitudes. In the LPP time window the ego depletion faking condition appears to be more similar to the baseline condition than compared to the ‘normal’ faking condition (see Fig. 4b). This finding could be related to a reduced ability to exert cognitive control in a state of ego depletion, as proposed by some researchers [34]. However, one should be aware that we found no differences in the faking *success* between both faking conditions. Since we regard shifts in the D-score as an indicator of faking success, we might even conclude that faking success was higher under ego depletion. To explain the difference between our behavioral and neuronal data, one might speculate that different brain activation patterns can lead to similar behavioral outcomes. This relates well to a recent review, which discusses evidence for compensatory brain activation under ego depletion [32]. However, another important reason for these differences is that ERPs and reaction times are essentially different measures. The ERP responses are direct measures of the processing of the presented visual stimuli. Here, next to the primary sensory processing, various other processing tasks modulate the ERP amplitudes, for example the response selection, response preparation and motor execution (and during faking an inhibition/delay of the response). On the other hand, reaction times are the very end of this process. Nevertheless, combining behavioral and neuronal measures might help to understand why sometimes differences are found under ego depletion, while sometimes no differences can be observed.

It should be mentioned, that in the absence of inhibitory tasks, slower responding has been found to affect late components like the P3 [60]. Here, smaller amplitudes are reported for faster compared to slower subjects [60]. Moreover, on the individual level, fast compared to slow responses were found to elicit a slightly earlier peaking P3 [60]. This complicates direct comparisons of our supposed faking blocks to the respective baseline block. Since we have a systematical reaction time difference between the faking and baseline doping + like blocks (as induced by the manipulation), this issue cannot be eliminated by an Analysis of Covariance [61]. However, first we do not see a delayed peaking P3 for the doping + like block during faking (cf. Fig. 4c). Second, since the reaction times were comparably slow in the two faking blocks, the differential pattern between ‘normal’ and depleted faking cannot be explained by the slower response or the execution of the button press. On the other hand, despite differences in response speed, the ERPs seem quite similarly going in the ego depletion and baseline condition. Finally, an overall comparable pattern can be observed already at the P1, not considered to be affected by slower responses.

In source space, an enhanced activity for faking in the bilateral middle temporal gyri/TPJ could be found within the ‘normal’ faking condition. This region has been reported to be more active during forced-choice faking [24]. However, as enhanced TPJ activity is also related to attention [62] and intentional action execution [63, 64] it has been previously interpreted to reflect the monitoring of the planned faking response [17]. However, TPJ activity is not restricted to attention and executive control. It has shown to play a role in a wide variety of domains such as theory of mind [65, 66], as well as in social cognition [67]. It has been suggested that the TPJ plays a role in both social cognitive specific as well as unspecific attention an memory processes [68]. No differences in activity were found for faking in right-inferior frontal regions [17], possibly due to a reduced signal to noise ratio for these estimations.

Source estimation results might also indicate a differential cerebral processing in the two faking conditions [32]. However, while for example Friese et al. [33] found decreased activity in the right lateral prefrontal cortex, we found no differences between the ego depletion faking and non-faking block. This might be due to the lower spatial resolution of the source estimations compared to fMRI measures. Depletion effects might lead to a reduction of activity within some parts of executive networks [32], although present source estimations may not be able to capture these changes. Further some studies suggest that under ego depletion increased activations can be found which are supposed to reflect compensatory effort [69, 70]. Although we did not find differences in source space on a reasonable threshold, compensatory activations within some parts of executive network might account for the discrepancy between ERPs for ‘normal’ and ego depleted faking.

On the other hand, in contrast to previously reported modulations of early frontal and occipital components [59], we did not find substantial differences, neither for depleted nor for ‘normal’ faking. Descriptively, participants in the ‘normal’ faking condition showed a negativity over frontal and positivity over occipital sensors in the supposed faking block (*doping* + *like*). However, the insignificant interaction prohibited post hoc testing. An explanation might be the increased difficulty of the task. So far, studies reporting such early effects have used a very simple faking task for the participants, providing an instruction *how* and *when* to fake [71]. So, by using such blocked designs participants could prepare how to respond, which might have affected even such early sensory processing. In the current experiment participants were not provided with an explicit instruction and had to find a strategy on their own. This could have led to unsystematic faking strategies. Thus, not all participants might have faked using a set response strategy. Some might have waited for the stimulus onset and *then* planed how to respond. In line with this interpretation, standard deviations seem to be larger in the supposed faking blocks than in previous study on instructed faking (instructed faking *SD* = 348 ms; ‘normal’ uninstructed faking *SD* = 587 ms; ego depletion uninstructed faking *SD* = 685 ms) [17]. Another explanation for smaller interactions is based on methodological reasons: In the previous study on IAT faking, participants were instructed to fake negative as well as positive doping attitudes, thus both faking conditions should deviate in a different direction from baseline [17]. In this experiment, the expected faking block and direction was the same for both faking conditions.

In principle, our results suggest that there is no specific ERP component for faking. In accordance with the literature [13–15, 18], we think that the cognitive processes involved in faking modulates the ERP responses. We have speculated that individual and temporal differences in the available self-control resources might affect the cerebral faking pattern. Supporting this, we found different EEG faking patterns for depleted and non-depleted participants. Future research on faking components might profit from monitoring both state and trait self-control as a possible moderator variable.

In regard to the BIAT, our study shows that it can be faked even if participants are not provided with an effective faking strategy and when their self-control resources are depleted (albeit to a lesser extent than when provided with an effective faking strategy; [17]). Instructing participants to fake an (B)IAT and even providing them with a faking strategy is the standard setup of IAT faking research [e.g., 8–10]. However, it has been argued that although this can be a powerful design to assess the principal fakeability of a test, it does not reflect real world faking [6]. Consequently, Wolff and colleagues [6] used an approach were participants were only implicitly incentivized to fake a BIAT: If their doping attitude—measured with a BIAT or a self-report measure—would exceed a certain (bogus) cut-off, participants would be subjected to a tedious anti-doping training program. This manipulation led to successfully faked scores only in the self-report measure but not in the BIAT. As such a setting is more likely to mirror real-world faking, it will be interesting to investigate the associated ERP patterns. Even more so in light of our finding that effectively faked scores in the ‘normal’ faking and the ego depleted faking condition are associated with different electro-cortical correlates. Thus, even though participants are not successful at faking a BIAT in an implicitly incentivized scenario, the ERP’s between the incentivized and the control condition may still differ. Finally, in cases were faking cannot be determined statistically from the behavioral data, ERPs and source reconstruction may help to distinguish between truth and lie.

## Conclusion

Although the effect is smaller, uninstructed BIAT faking in normal state resembles previous results from instructed BIAT faking, both behaviorally and neurophysiologically. However, our results also indicate that while participants succeed in faking a BIAT when self-control resources are temporarily depleted, the associated ERP patterns differ. When depleted, ERPs differences at early and late processing stages are similar to the baseline pattern but different from ‘normal’ faking results. In contrast to ‘normal’ faking, no reliable sources were found for faking under ego depletion. Taken together, our results underline the importance of further understanding the electrophysiological correlates of test faking: The differential effect on brain and behavior may help to distinguish between faking and honest responding in ecologically more valid experimental setups.

## Additional files

10.1186/s12868-016-0249-8 Legend to datafile.
10.1186/s12868-016-0249-8 Comma delimited datafile.
10.1186/s12868-016-0249-8 Datafile.
10.1186/s12868-016-0249-8 Significant interaction between block and condition.

## Abbreviations

BEM: boundary element head model
BIAT: brief implicit association test
CMS: common mode sense active electrode
DRL: driven right leg passive electrode
EEG: electroencephalography
ERN: error related negativity
ERP: event related potential
fMRI: functional magnetic resonance imaging
IAT: implicit association test
LPP: late positive potential
PANAS: positive and negative affect schedule
TPJ: temporo-parietal junction
