# Supplementary figures and images for: Uninstructed BIAT faking when ego depleted or in normal state: differential effect on brain and behavior
Source: BMC Neurosci. 2016 May 3;17:18. doi: 10.1186/s12868-016-0249-8 (PMC4855370; doi:10.1186/s12868-016-0249-8)

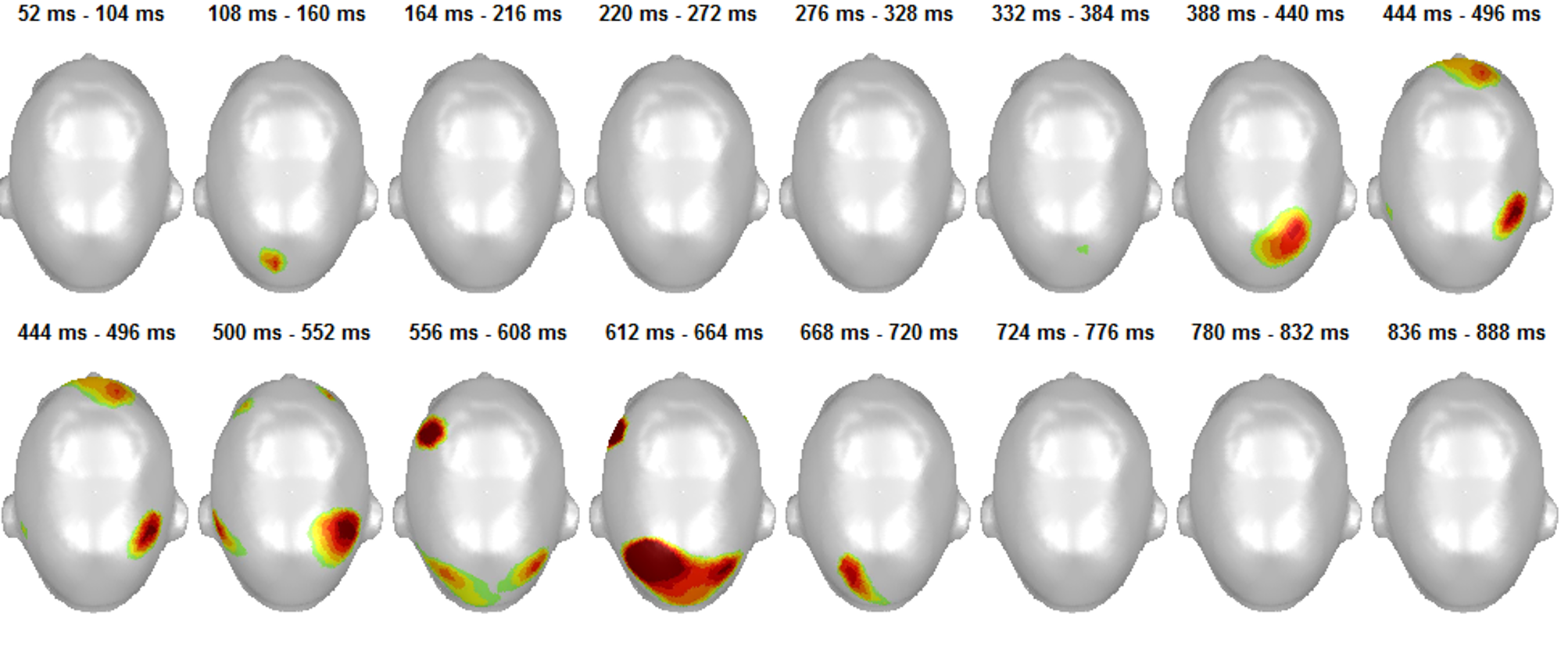

Supplement: Supplementary file 4 — 10.1186/s12868-016-0249-8 Significant interaction between block and condition. [file 12868_2016_249_MOESM4_ESM.tiff]
